# The Pressing Need to Raise Awareness about Osteoarthritis Care among Elderly Females in Pakistan: A Cross-sectional Study

**DOI:** 10.7759/cureus.5302

**Published:** 2019-08-01

**Authors:** Fatima Saeed, Amal Humayun, Syeda M Fatima, Vashma Junaid, Hooria Imtiaz, Maheen Zehra, Amna Zahid, Ayesha Channa, Anjiya I Meherally, Zunaira Z Shah, Amreen Hoosseny, Aiman Khurshid, Salman Tariq, Samar Mahmood, Kaneez Fatima

**Affiliations:** 1 Medicine, United Medical and Dental College, Karachi, PAK; 2 Internal Medicine, Ziauddin Medical University, Karachi, PAK; 3 Internal Medicine, Dow University of Health Sciences, Karachi, PAK; 4 Medicine, Civil Hospital, Dow University of Health Sciences, Karachi, PAK; 5 Medical Student, Ziauddin Medical University, Karachi, PAK; 6 Internal Medicine, Jinnah Sindh Medical University, Karachi, PAK; 7 Internal Medicine, Dow Medical College and Civil Hospital, Karachi, PAK

**Keywords:** osteoarthritis, knowledge, elderly females, joint pain, osteoarthritis awareness, osteoarthritis self care, pakistan, public awareness, pakistani females

## Abstract

Introduction

Osteoarthritis (OA) is the single-most common cause of physical disability among adults. In view of its promising management modalities, an analysis of the level of awareness among the suspected individuals and their attitudes is crucial to assess the level of their implementation. This study aimed to do that among the female population in Karachi, Pakistan.

Methods

This cross-sectional study was conducted among 316 elderly females (≥60 years) in Karachi in 2018. Data were collected via a structured questionnaire, which included sections titled: demographics, knowledge, attitudes, and practices. Data were analyzed using the Statistical Package for Social Sciences (version 20.20, IBM, Armonk, New York, US), and chi-square tests were used to assess the connection between OA care and socioeconomic statuses. Mean and standard deviation were calculated for quantitative variables.

Results

A considerable portion (48%, n = 152) of the participants were from a lower socioeconomic background, and 51% of them had a history of joint pain. Significantly, 63% of the participants (n = 199) attributed their joint pain to age, while nearly half attributed it to their diet and exercise habits. A major segment (73%, n = 230) of the participants, irrespective of their socioeconomic backgrounds, had visited a doctor for their joint complaints. Around 65% of the participants said they would never undergo a knee-joint replacement, regardless of how worse their symptoms might get. Additionally, 36% of the participants were unsatisfied with their current treatment, while more than half of the participants said that medication would improve their condition.

Conclusions

Our results demonstrate a connection between the lack of awareness about OA and the factors negatively affecting its management. They also point towards areas that require focused efforts for better awareness.

## Introduction

Osteoarthritis (OA) is a degenerative disease of the joints that is characterized by the deterioration of the cartilage lining them, causing the bones to rub against each other. It creates a painful, stiff joint disability with decreased motion [1]. It is reported to be the single-most common cause of physical disability among adults [2], notably impacting the hip and knees. It is estimated to affect 18% of females and 9.6% of males above the age of 60 years worldwide [3]. By 2040, more than 16% of Asians are predicted to be 65 years old [4], and 78.4 million among them are projected to be affected by OA [5]. Although OA is most commonly associated with advancing age, other factors also play a role in its development, such as obesity, lack of exercise, and genetic predisposition. It is more commonly associated with females than males [6]. The Global Burden of Disease Study conducted in 2013 showed the prevalence of OA to be 26.67 per 1,000 people in Pakistan [7]. In a cross-sectional study conducted at a tertiary-care hospital in Karachi, the prevalence of OA was found to be 7.35% among 346 diagnosed cases of rheumatic disorders [8].

Management strategies targeted at OA care recommend both non-pharmacological and pharmacological therapies. Non-pharmacological approaches such as lifestyle changes, patient education, self-management, and physical therapy have been reported to improve quality of life and reduce pain and disability in OA patients. Therapy by medication, such as acetaminophen and non-steroidal anti-inflammatory drugs (NSAIDs) was found to be more effective when used alongside non-pharmacological strategies [9]. Studies have shown that physical exercise helps reduce physical dependence and disability among OA patients [10].

Given the evidence in favor of promising management modalities for OA, an analysis of the awareness and attitudes of the suspected and suffering individuals is necessary to assess the level of implementation of the effective modalities. This study aimed to address this gap by addressing elderly women’s knowledge of OA in Karachi, their attitude towards the management of the disease, and the practices adopted by them for its care. By investigating the behavior of these women, we aimed to devise implementable methods to fill in the knowledge gaps and improve the overall health practices of OA patients. Subsequently, we aimed to reduce the physical disability caused by the condition, decrease the economic burden that the disease poses nationally and internationally, and improve quality of life at large.

## Materials and methods

Sample and setting

This cross-sectional study was conducted among elderly females in Karachi, on approval from the Institutional Review Board (IRB) of the Dow University of Health Sciences in Karachi. The selected sample consisted of women who were 60 years and above of age and had complaints of bone/joint pain. Women from all socioeconomic backgrounds with any other associated co-morbidities were also included, as were those with or without a family history of joint complaints and those who were known cases of OA. Females below 60 years of age and those in the premenopausal phase were excluded, along with those who had congenital problems. The sampling technique used was that of convenience sampling. The required sample size arrived at was of 316 subjects, which was calculated using OpenEpi program (version 3.01, Rollins School of Public Health, Emory University, Atlanta, Georgia, US). This enabled us to fulfill the objectives of our study at 95% confidence level and with a margin of error of 5%. A total of 351 women were interviewed, leading to an eventual data set of 316 participants and a cooperation rate of 90%. The data were collected from different areas in Karachi over a five-month period (May-October, 2018).

Data collection

Data were collected by a group of well-trained researchers. They conducted interviews based on a self-structured questionnaire and the results were recorded accordingly. The questionnaire was divided into four sections: demographics, knowledge, attitudes, and practices. The demographics covered queries about age, weight, occupation, socioeconomic status, and family-related history of joint pain. Other associated modifiable and non-modifiable risk factors like diabetes and hypertension were also covered. Knowledge was assessed among the sample population to see if they had attended any awareness program(s) regarding joint pain. The patients’ understanding of the causes of joint pain, the role of pain-killer medications in curing it, and their potential need to have surgery to implant artificial joints in the future were assessed. The attitude section included questions about multidimensional therapies, the role of massaging, causes for visiting a doctor for joint pain, and the association of joint pain to a disease. It also dealt with the intake of medicines, impact of joint pain on social life, frequency of changing a physician, patients' beliefs regarding the progression of the joint pain, and satisfaction with the current approach. Lastly, the practices section covered the relevance of compliance with medications, diagnostic screening tests and their evaluation. The section also addressed the role of physical activities, exercise, dietary intake and supplementation.

A pilot was run among 30 participants, prior to the actual data collection, and the feedback from these participants was used to modify the questionnaire to improve comprehension.

Data were analyzed using Statistical Package for Social Sciences (version 20.20, IBM, Armonk, New York, US) and assessed qualitatively. For quantitative variables like age, weight, and height, mean and standard deviation were calculated.

Reduction of bias

Questions concerning only recent happenings or lifestyle norms were used to minimize the recall bias. Uniformly trained data collectors were used to collect data to prevent interviewers’ bias. All patients were given an equal time to answer the questions and no leading questions were asked. Utmost care was taken to avoid assumptions made on the basis of appearance or socioeconomic background. Further, the Berksonian bias was eliminated by including only women with joint pain.

## Results

**Figure 1:**
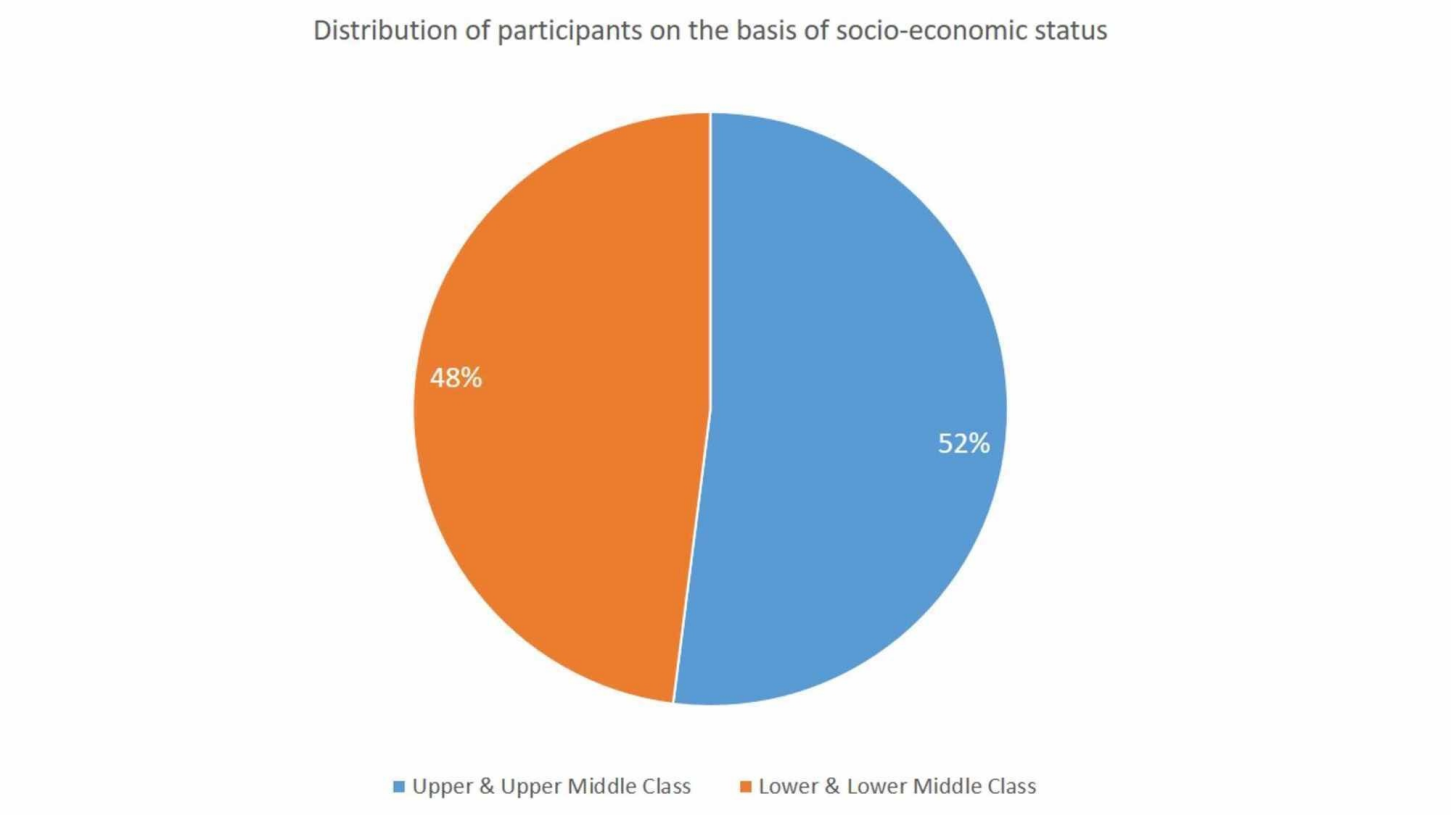
Distribution on the basis of socioeconomic status.

The mean weight of the participants was 68.3 kg (±12.5 kg), while the average height was 5 ft (±0.2 ft). A considerable majority of the participants people were from a lower socioeconomic background, and 96% (n = 157) of the people from the upper class hailed from families with less than five earning members (Figure 1).

Of those belonging to the upper and upper-middle class, 64% (n = 104) had a history of joint pain in the family, while 51% (n = 77) of those belonging to the lower and lower-middle class had a similar history. Out of the 316 participants, 63% (n = 199) attributed their joint pain to age, while nearly half also attributed it to their diet and exercise habits; 15% (n = 48) believed the reason to be genetic as well.

A notable 65% (n = 208) of the participants said they would never undergo a knee-joint replacement, regardless of how worse their symptoms might get. And 35% (n = 110) the participants who expressed such a view belonged to the lower economic background. Around 76% (n = 240) of the participants, irrespective of their socioeconomic backgrounds, said that massaging helped relieve their pain for a short period of time. And 43% (n = 135) of the participants believed that their joint problem was a disease.

A major portion 73% (n = 230) of the participants, irrespective of economic backgrounds, had visited a doctor for their joint complaints. Around 186 participants (59%) said that joint pain was the most important driving factor for them to visit a doctor, while a third believed that physical inactivity was the most important cause of their joint pain. More than half of the participants said that their symptoms had affected their social lives either moderately or severely. Additionally, 36% (n = 114) of all of the participants were unsatisfied with their current approach to treatment and believed that their symptoms would get worse in the upcoming years. More than half (57%, n = 180) of the participants said that taking medications would improve their condition. NSAIDs and paracetamol were reported to be the most common category of medications taken by them.

Only 30% (n = 94) of the participants said they exercised or took part in any form of physical activity on a regular basis. The most common supplements taken by the majority of participants in both categories were vitamin D, calcium, and vitamin B12. And 237 (75%) participants said they were exposed to sunlight on a daily to weekly basis.

**Figure 2:**
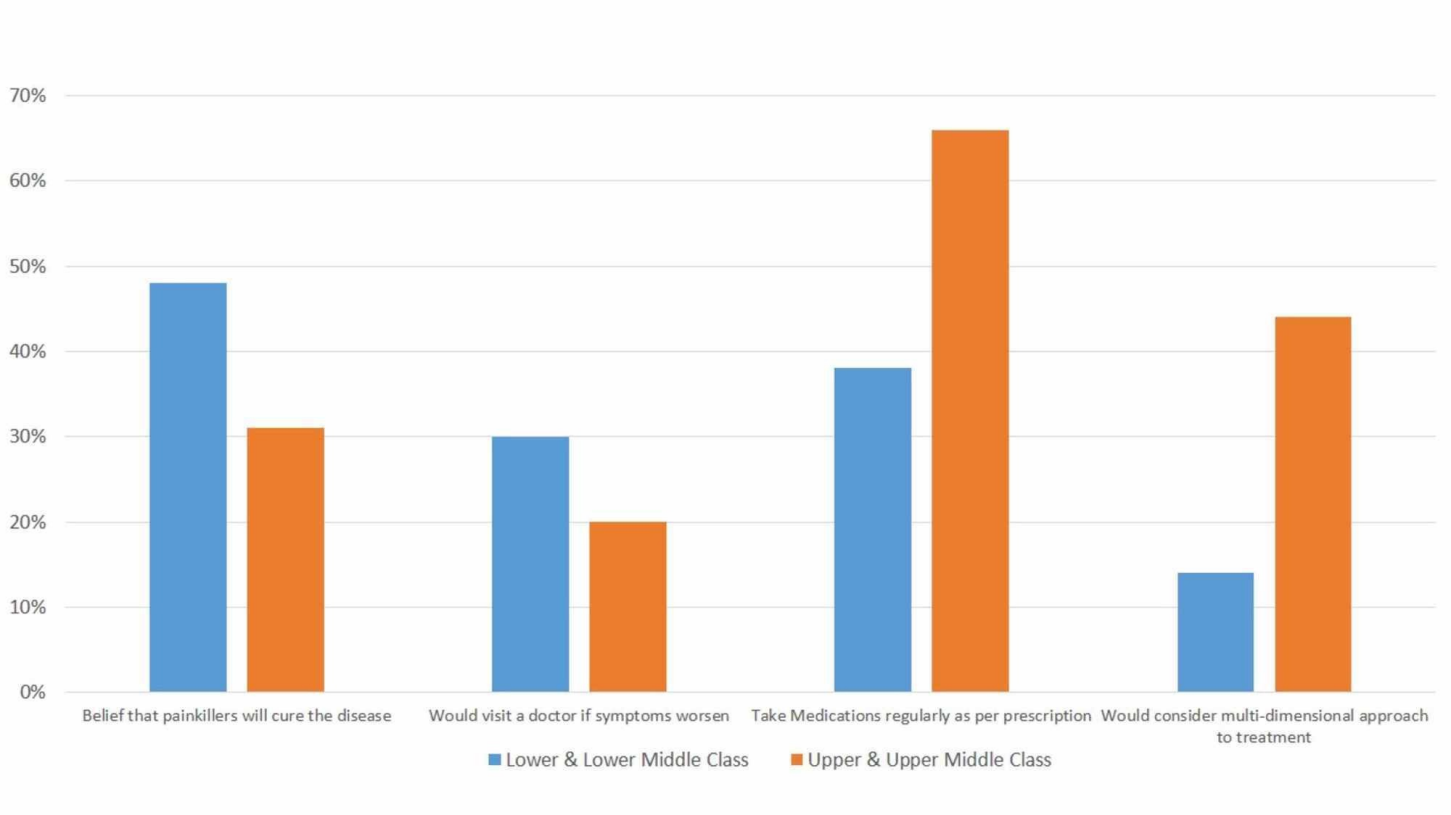
Comparison between the knowledge, attitudes, and practices of the upper and lower classes regarding osteoarthritis care.

**Figure 3:**
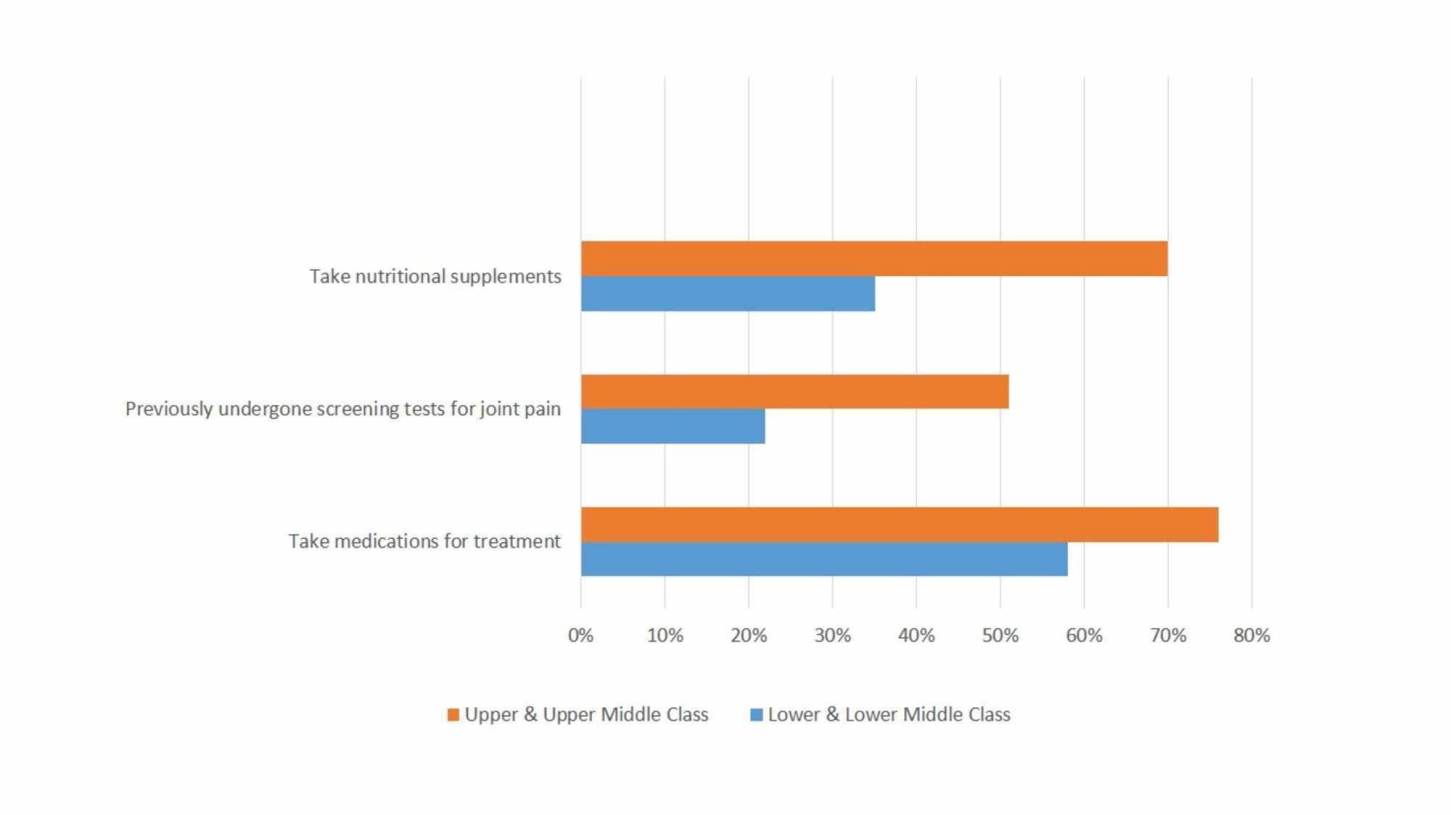
Comparison between the practices of the upper and lower classes regarding osteoarthritis care.

Table 1 shows the percentage of participants in each category who consumed red meat, processed foods, soft drinks, fruits and vegetables, and whole grains on a weekly to monthly basis. Figures 2 and 3 compare knowledge, attitudes, and practices in the sample population on the basis of their socioeconomic status.

**Table 1:** Participants’ consumption of dietary substances based on their socioeconomic background.

	Lower and lower middle class (%)	Upper and upper middle class (%)
Red meat	31	22
Processed foods	20	31
Soft drinks	29	35
Fruits, vegetables, and whole grains	80	95

## Discussion

The findings of our cross-sectional study largely demonstrate a lack of awareness about OA and its management among our sample population. With 63% (n = 199) of the participants attributing their joint pain to age, nearly a half attributing it to lack of exercise and diet, and about 15% (n = 47) believing its cause to be genetic, the huge deficit in the knowledge regarding OA care became fairly apparent.

In the past studies done on the topic, the main objective of the treatment was touted to be to minimize the pain and ameliorate the functional capacity of the patients. There has also been an increasing need for an emphasis on non-operative management such as patient education, exercise therapy, oral dietary supplements, and walking programs [11]. In order to promote these methods, it is very important to work on the patients’ attitudes towards the conservative management of the disease and help them understand it in a better way.

Moreover, preventing obesity and sustaining lean body mass are fundamental to improving aging-related musculoskeletal changes [12]. The American College of Rheumatology (ACR), the European League Against Rheumatism (EULAR), and the Osteoarthritis Research Society International (OARSI) suggest exercise as vital in OA care [9, 13-14]. However, only 30% (n = 94) of the participants from our study said they exercised or took part in physical activity on a daily basis, demonstrating a lack of knowledge regarding management of OA. Better communication with healthcare professionals and initiating awareness programs can help to bridge this gap and empower patients to feel in control of the management of their condition. OA could also be better treated in Pakistan if detailed treatment plans are made widely and cheaply available to women of all classes. Devising OA management also requires the importance of exercise to be emphasized and made a part of lifestyles early on in life [15].

Previous studies have shown that the complications of OA not only include physical problems, but it also has unfavorable psychological effects. As compared to patients with other chronic diseases such as diabetes, psychological distress is more commonly experienced by patients with OA [16]. Our study showed that around 40% of women from the upper class and around 10% from the lower class were willing to consider multidimensional treatments like physiotherapy, psychiatry and occupational therapy. This finding, in addition to being indicative of how their respective financial resources and awareness levels impact their healthcare choices, also exhibits how there is room for physicians to educate other patients to help change their attitude towards the disease. This would enable them to make informed decisions about the treatments.

In addition, the literature states that a weight loss of greater than 10%, over a period of four years, slows knee cartilage degeneration [17]. However, the mean weight of the participants interviewed in our study was 68.3 kg (±12.5 kg), proving the lack of awareness about the importance of weight loss in the management of OA in the elderly. This could be attributed to people relating the joint pain that comes with movement and exercise with worsening of the disease and consequently refraining from most physical activities that in the long term would actually improve their quality of life. This draws attention to another misbelief that warrants correction. According to our study, only 51% (n = 83) of the upper class and just 22% (n = 33) of the lower class participants would consider going for screening tests for an early diagnosis. This could be due to financial constraints and a lack of awareness that their condition is due to a disease process. Clinical OA is a late-stage condition that has very restricted treatment options. But the fact that it takes decades to develop can help us to potentially modify the course of the disease with early diagnosis. The surgeon’s assessment of the articular-cartilage softening observed in early screening denotes the earliest clinical sign that is detectable for pre-OA known as chondrosis or chondromalacia [18]. The basic qMRI techniques studied in pre-OA screening include delayed gadolinium-enhanced MRI of the cartilage (dGEMRIC) [19], T2 and T1rho mapping [20], and ultrashort echo-time enhanced T2* (UTE-T2*) mapping [21]. While subjective, most screening methods play a very important role in managing the course of the disease.

Furthermore, our findings showed that 208 (65%) participants said they would never undergo a knee-joint replacement, regardless of how worse their symptoms might get. The available data on the success rate remains vague in Pakistan. This means that elderly women who are already distressed due to their health are even less likely to consider it as a treatment option. Untreated risk factors like obesity and advanced age in post-op patients of arthroplasty and other surgeries may explain the general pattern of failed surgeries. Active methods must be undertaken to alleviate the misconceptions regarding knee surgeries, the greatest one being that it worsens the joint’s health [22].

There are several limitations in this study that need to be mentioned. Firstly, the sample size was relatively small. However, it represented a population of varied socioeconomic levels, which helped us gain an all-rounded picture of the variations in awareness levels and attitudes. Secondly, the study was based on the practices of elderly women only. Future researchers would possibly depict the awareness and attitudes better if they included individuals of both the sexes and all ages for more accurate results. Further, co-morbidities were not considered in the exclusion criteria.

## Conclusions

Our results demonstrate a connection between the lack of awareness about OA care and the factors negatively affecting its management. The ability to detect pre-OA symptoms prior to the onset of irreversible changes is crucial for understanding the disease process, though a significant number of participants in our study said they would not consider getting screened for OA. We also strive to highlight that areas that need more focus are improved patient awareness and attitudes about OA. We also discuss avenues where further research can be conducted.

## References

[1] (2019). What is osteoarthritis?. https://www.arthritis.org/about-arthritis/types/osteoarthritis/what-is-osteoarthritis.php.

[2] Ettinger WH Jr, Fried LP, Harris T, Shemanski L, Schulz R, Robbins J (1994). Self-reported causes of physical disability in older people: the cardiovascular health study. CHS Collaborative Research Group. J Am Geriatr Soc.

[3] (2019). Chronic rheumatic conditions. https://www.who.int/chp/topics/rheumatic/en/.

[4] Fransen M, Bridgett L, March L, Hoy D, Penserga E, Brooks P (2011). The epidemiology of osteoarthritis in Asia. Int J Rheum Dis.

[5] Hootman JM, Helmick CG, Barbour KE, Theis KA, Boring MA (2016). Updated projected prevalence of self-reported doctor-diagnosed arthritis and arthritis-attributable activity limitation among US adults, 2015-2040. Arthritis Rheumatol.

[6] Haq I, Murphy E, Dacre J (2003). Osteoarthritis. Postgrad Med J.

[7] Moradi-Lakeh M, Forouzanfar MH, Vollset SE (2017). Burden of musculoskeletal disorders in the Eastern Mediterranean region, 1990-2013: findings from the global burden of disease study 2013. Ann Rheum Dis.

[8] Mohsin Z, Asghar AA, Faiq A (2018). Prevalence of rheumatic diseases in a tertiary care hospital of Karachi. Cureus.

[9] Hochberg MC, Altman RD, April KT (2012). American College of Rheumatology 2012 recommendations for the use of nonpharmacologic and pharmacologic therapies in osteoarthritis of the hand, hip, and knee. Arthritis Care Res (Hoboken).

[10] Van Gool CH, Penninx BW, Kempen GI (2005). Effects of exercise adherence on physical function among overweight older adults with knee osteoarthritis. Arthritis Rheum.

[11] Buckwalter JA, Stanish WD, Rosier RN, Schenck RC Jr, Dennis DA, Coutts RD (2001). The increasing need for nonoperative treatment of patients with osteoarthritis. Clin Orthop Relat Res.

[12] Fielding RA (1995). The role of progressive resistance training and nutrition in the preservation of lean body mass in the elderly. J Am Coll Nutr.

[13] Fernandes L, Hagen KB, Bijlsma JW (2013). EULAR recommendations for the nonpharmacological core management of hip and knee osteoarthritis. Ann Rheum Dis.

[14] McAlindon TE, Bannuru RR, Sullivan MC (2014). OARSI guidelines for the nonsurgical management of knee osteoarthritis. Osteoarthritis Cartilage.

[15] Garver MJ, Focht BC, Taylor SJ (2015). Integrating lifestyle approaches into osteoarthritis care. J Multidiscip Healthc.

[16] Penninx BW1, Beekman AT, Ormel J, Kriegsman DM, Boeke AJ, van Eijk JT, Deeg DJ (1996). Psychological status among elderly people with chronic diseases: does type of disease play a part?. J Psychosom Res.

[17] Gersing AS, Solka M, Joseph GB (2016). Progression of cartilage degeneration and clinical symptoms in obese and overweight individuals is dependent on the amount of weight loss: 48-month data from the osteoarthritis initiative. Osteoarthritis Cartilage.

[18] Outerbridge RE (1961). The etiology of chondromalacia patellae. J Bone Joint Surg Br.

[19] Owman H, Tiderius CJ, Neuman P, Nyquist F, Dahlberg LE (2008). Association between findings on delayed gadolinium-enhanced magnetic resonance imaging of cartilage and future knee osteoarthritis. Arthritis Rheum.

[20] Hovis KK, Stehling C, Souza RB (2011). Physical activity is associated with magnetic resonance imaging-based knee cartilage T2 measurements in asymptomatic subjects with and those without osteoarthritis risk factors. Arthritis Rheum.

[21] Du J, Takahashi AM, Chung CB (2009). Ultrashort TE spectroscopic imaging (UTESI): application to the imaging of short T2 relaxation tissues in the musculoskeletal system. J Magn Reson Imaging.

[22] Salih S, Sutton P (2013). Obesity, knee osteoarthritis and knee arthroplasty: a review. BMC Sports Sci Med Rehabil.

